# Diosgenin ameliorates palmitic acid-induced lipid accumulation via AMPK/ACC/CPT-1A and SREBP-1c/FAS signaling pathways in LO2 cells

**DOI:** 10.1186/s12906-019-2671-9

**Published:** 2019-09-13

**Authors:** Ke Fang, Fan Wu, Guang Chen, Hui Dong, Jingbin Li, Yan Zhao, Lijun Xu, Xin Zou, Fuer Lu

**Affiliations:** 1 Department of Integrated Traditional Chinese and Western Medicine, Tongji Hospital, Tongji Medical College, Huazhong University of Science and Technology, Wuhan, 430030 China; 2 Institute of Integrated Traditional Chinese and Western Medicine, Tongji Hospital, Tongji Medical College, Huazhong University of Science and Technology, Wuhan, 430030 China

**Keywords:** Diosgenin, Nonalcoholic fatty liver disease (NAFLD), AMPK/ACC/CPT-1A, SREBP-1c/FAS

## Abstract

**Background:**

Non-alcoholic fatty liver disease (NAFLD) is the most common chronic liver disease and is characterized by excessive hepatic lipid accumulation. Many studies have suggested that lipid overload is the key initial factor that contributes to hepatic steatosis. Our previous study indicated that diosgenin (DSG) has a beneficial effect on energy metabolism, but the underlying mechanism remains unclear.

**Methods:**

Human normal hepatocytes (LO2 cells) were incubated with palmitic acid to establish the cell model of nonalcoholic fatty liver. The effects of DSG on lipid metabolism, glucose uptake and mitochondrial function were evaluated. Furthermore, the mechanism of DSG on oxidative stress, lipid consumption and lipid synthesis in LO2 cells was investigated.

**Results:**

The results indicated that palmitic acid induced obvious lipid accumulation in LO2 cells and that DSG treatment significantly reduced the intracellular lipid content. DSG treatment upregulated expression of lipolysis proteins, including phospho-AMP activated protein kinase (p-AMPK), phospho-acetyl-coA carboxylase (p-ACC) and carnitine acyl transferase 1A (CPT-1A), and inhibited expression of lipid synthesis-related proteins, including sterol regulatory element-binding protein 1c (SREBP-1c) and fatty acid synthase (FAS). Additionally, DSG-treated cells displayed a marked improvement in mitochondrial function, with less production of reactive oxygen species and a higher mitochondrial membrane potential compared with the model group.

**Conclusion:**

This study suggests that DSG can reduce intracellular lipid accumulation in LO2 cells and that the underlying mechanism may be related to the improving oxidative stress, increasing fatty acid β-oxidation and decreasing lipid synthesis. The above changes might be mediated by the activation of the AMPK/ACC/CPT-1A pathway and inhibition of the SREBP-1c/FAS pathway.

## Background

With a rapid increase in prevalence, NAFLD has become the most common chronic liver disease in the world [1]. NAFLD is characterized by excessive hepatic lipid accumulation and metabolic dysfunction. According to its pathological features, NAFLD can be divided into different subgroups, including nonalcoholic fatty liver (NAFL), nonalcoholic steatohepatitis (NASH) and associated cirrhosis. The “double-hit” model is the most widely accepted hypothesis to explain the pathogenesis of NAFLD. The “first hit” is induced by fat accumulation in the hepatocytes and insulin resistance, which increases the “sensitization” of the “second hit” by various pro-inflammatory mediators, eventually leading to hepatocyte damage and inflammation [2]. Many studies have suggested that lipid overload is the key factor contributing to hepatic steatosis [3, 4]. Therefore, protecting hepatocytes from lipid accumulation is an important strategy to prevent and treat NAFLD.

Lipid levels in the liver are dynamically balanced between lipid synthesis and lipid consumption, and unbalanced lipid metabolism induces excessive lipid accumulation in the liver and leads to hepatic steatosis. Therefore, accelerating lipid consumption and/or inhibiting lipid synthesis is considered an effective solution to reduce hepatic lipid accumulation [5]. Mitochondrial fatty acid β-oxidation (FAO) is a major route of lipid consumption in which free fatty acids (FFA) are esterified with CoA, transported into the mitochondria matrix, and oxidized to generate acetyl-CoA, which finally enters β-oxidation [6]. Many studies focused on activating liver FAO have suggested a potential approach to treat metabolic disorders, such as NAFLD and diabetes [4, 7, 8]. In FAO, the carnitine palmitoyl transferase (CPT) system mediates the transport of FFAs; this system consists of three proteins: CPT1, CPT2, and acylcarnitine translocase. CPT1 is responsible for the initial enzymatic reaction for FFA transport and is also considered as the rate-limiting enzyme for FAO. The activation of CPT1 promotes energy expenditure and has metabolic benefits [6, 9]. It is well known that mitochondrial dysfunction contributes to the pathogenesis of NAFLD because it affects hepatic lipid homeostasis and promotes lipid peroxidation, cytokine release and cell death [10]. Accumulating evidence suggests that improving mitochondrial dysfunction is an effective strategy for reducing lipid overload. On the other hand, de novo lipogenesis in the liver greatly contributes to hepatic steatosis, and inhibition of fatty acid synthesis has been proven to be beneficial for NAFLD mice [3]. Altogether, evidence indicates that promoting FAO and inhibiting fatty acid synthesis under the condition of hepatic lipid accumulation are of substantial interest.

AMP-activated protein kinase (AMPK) is believed to play a central role in controlling lipid metabolism by modulating the downstream acetyl-CoA carboxylase (ACC) and CPT1 pathway [11]. ACC catalyzes the production of malonyl-CoA, which is both a major building block for de novo lipogenesis and an allosteric inhibitor of CPT1 [12]. The phosphorylation of ACC by AMPK leads to the inactivation of ACC, which is beneficial for the recovery of CPT-1 activity and FAO. Many studies have shown that the activation of the AMPK/ACC/CPT-1A pathway promotes free fatty acid oxidation [13, 14]. On the other hand, sterol regulatory element-binding protein 1c (SREBP-1c), a major regulator of lipogenic proteins such as fatty acid synthase (FAS), and its activity are also under the control of AMPK. In NAFLD mice, the activity of the SREBP-1c/FAS pathway was shown to be obviously elevated [15], and to contribute to the progression of hepatic steatosis. Li et al. showed that inhibiting SREBP-1c activity can exert an anti-hepatic steatosis effect in diet-induced obese mice [3]. The correlation between inhibition of the SREBP-1c/FAS pathway and decreased liver lipid synthesis has been well established [16–18]. Therefore, the activation of the AMPK/ACC/CPT-1A pathway and inhibition of the SREBP-1c/FAS pathways may be potential targets for screening drugs to prevent hepatic lipid accumulation.

Diosgenin (DSG), an active component isolated from *Trigonella foenum-graecum* (Hu-Lu-Ba in Chinese), is used to treat metabolic disorders in traditional Chinese medicine [19, 20]. Our previous study showed that DSG can ameliorate insulin resistance in HepG2 cells [21], suggesting that it affects lipid catabolism. Although the beneficial effects of DSG on energy metabolism have been identified, its molecular mechanisms are still unclear, especially in lipid consumption [22]. In this study, we established a cell model of NAFLD by palmitic acid (PA)-induced lipid accumulation in LO2 cells and investigated the effect and underlying mechanism of DSG in lipid metabolism in vitro.

## Methods

### Chemicals and reagents

Fetal bovine serum (FBS) was obtained from Biological Industries Israel Beit Haemek Ltd. (Israel). Dulbecco’s modified Eagle’s medium (DMEM) was purchased from Thermo Fisher Scientific Co. (USA). DSG was purchased from Aoke Biology Research Co. Ltd. (Beijing, China). Palmitate (PA) and Oil Red O dye were obtained from Sigma-Aldrich Co. (St. Louis, MO, USA). Compound C and A-769662 were provided by Selleck Co. (Shanghai, China). 2′,7′-Dichlorodihydrofluorescein diacetate (DCFH-DA) and JC-1 were obtained from Beyotime Institute of Biotechnology (Shanghai, China). Trypsin, penicillin, streptomycin, the Western blot kit, 4′,6-diamidino-2-phenylindole (DAPI), Triton X-100, and the Bicinchoninic acid (BCA) protein assay kit were purchased from Guge Biological Technology Co. (Wuhan, China). Cell Counting Kit-8 was provided by Dojindo Laboratories (Kumamoto, Japan). 2-(N-(7-nitrobenz-2-oxa-1,3-diazol-4-yl) amino)-2-deoxyglucose (2-NBDG) was obtained from Cayman Chemical Co. (USA). The triglyceride assay kits and other biochemical assay kits were purchased from Nanjing Jiancheng Bioengineering Institute (Nanjing, China). The rabbit AMP activated protein kinase (AMPK), rabbit phospho-AMP activated protein kinase (p-AMPK), rabbit acetyl-coA carboxylase (ACC), rabbit phospho-acetyl-coA carboxylase (p-ACC), rabbit carnitine acyl transferase 1A (CPT-1A), mouse fatty acid synthase (FAS) and rabbit Cytochrome c (Cyc) antibodies were from Cell Signaling Technology (MA, USA). The mouse monoclonal antibody against sterol regulatory element-binding protein 1c (SREBP-1c) was purchased from Santa Cruz Biotechnology (Santa Cruz, CA, USA). The DyLight™ 800 4X PEG Conjugated goat anti-mouse and goat anti-rabbit second antibodies were provided by Jackson Labs Technologies, Inc. (NV, USA). All other reagents were obtained from Biosci Biotechnology Co. Ltd. (Wuhan, Hubei, China) unless otherwise specified.

### Cell culture, viability assay and treatment

LO2 cells were provided by the Department of Hepatic Surgery, Tongji hospital, Huazhong University of Science and Technology. The cells were cultured in DMEM medium supplemented with 10% FBS, 100 units/mL penicillin, and 100 μg/mL streptomycin and maintained at 37 °C in a humidified atmosphere of 5% CO_2_ and 95% air. Cell viability was determined by the CCK-8 assay according to the manufacturer’s protocol.

Briefly, LO2 cells were seeded at 1 × 10^4^ cells/well in 96 well-culture plates. The medium was replaced with DMEM containing different concentrations of chemicals after cells had adhered; the cells were then incubated for another 24 h. The treatment medium was removed and replaced with 100 μL CCK-8 working solution. Then, the cells were incubated at 37 °C for 1 h. The absorbance at 450 nm was measured on a Synergr2 multifunctional microplate reader (Bio-Tek, USA).

LO2 cells were seeded at 3 × 10^5^ cells/well in 6 well-culture plates and allowed to grow overnight to 70% confluence. To induce lipid accumulation, PA was added to the medium and incubated for 24 h. The cells were divided into different groups as follows: (1) control group (incubated in DMEM containing 10% FBS), (2) model group (PA at a selective concentration for 24 h), (3) DSG groups (PA + different concentrations of DSG for 24 h), (4) AMPK activator group (PA + A-769662 for 24 h), and (5) AMPK inhibitor group (PA + DSG + Compound C for 24 h). In the AMPK inhibitor group, cells were pre-incubated with Compound C for 5 h to inhibit the activation of AMPK in advance, and then treated with PA and DSG for another 24 h.

### Oil red O staining

The cellular lipid content was determined by Oil Red O staining. Cells were washed with PBS twice and fixed in 4% paraformaldehyde for 30 min, and incubated with Oil Red O working solution for another 30 min at room temperature, followed by decolorization with 60% isopropanol. After washing three times with PBS, the cells were counterstained with hematoxylin for 30s. The cells were photographed under a light microscope (Olympus, Japan).

### Biochemical measurements

After 24 h of treatment, cells were collected and broken using an ultrasonic cell disrupter system (Sonics & Materials, Newton, CT, USA). The intracellular triglyceride (TG) content was measured using a triglyceride assay kit. Glutathione peroxidase (GSH-PX), superoxide dismutase (SOD), catalase (CAT) and malonaldehyde (MDA) were determined using the corresponding assay kits. To determine the levels of glutamic-pyruvic transaminase (ALT), glutamic oxalacetic transaminase (AST) and γ-glutamyl transpeptidase (γ-GT), the cell supernatant was measured using the corresponding assay kits. All the experiments were performed according to the manufacturer’s instructions.

### Measurement of glucose uptake

2-NBDG is a fluorescent deoxyglucose analog that can be taken up by cells but cannot be fully utilized and therefore accumulates inside cells. By measuring the fluorescence intensity, we can estimate the glucose uptake levels of LO2 cells. Briefly, cells were seeded at 1 × 10^4^ cells/well in 96 well-culture plates. The intervention was the same as before. Thereafter, the medium was removed and then washed twice with PBS. Then, 100 μL glucose-free DMEM containing 100 nM insulin and 200 μM 2-NBDG was added to the plates for 60 min. The fluorescence intensity at 528 nm was measured on a Synergr2 multifunctional microplate reader (Bio-Tek, USA) after washing twice with pre-cold PBS.

### Western blot analysis

To extract cell proteins, LO2 cells were lysed at 4 °C with RIPA lysis buffer containing phenylmethanesulfonyl fluoride (PMSF) and a protease inhibitor cocktail. After centrifugation at 12,000 g for 15 min at 4 °C, the supernatants were assayed to determine the protein concentration using the BCA method. Fifty μg protein was solubilized in SDS loading buffer and heated in boiling water for 10 min; then it was separated on 10% SDS-PAGE (120 v, 90 min) and transferred onto nitrocellulose (NC) membranes (280 mA). The membranes then were blocked with 5% BSA powder in ultrapure water for 1 h, followed by incubation with primary antibodies (p-AMPK, AMPK, p-ACC, ACC, CPT1A, SREBP-1c, FAS, Cyc and β-actin) overnight at 4 °C. Wash the membranes with TBST three times for 10 min each and incubate the membranes with fluorescence-labeled secondary antibodies for 1 h at room temperature. After another three washes, the membranes were detected by a near-infrared fluorescence imaging system (Odyssey, Lincoln, NB, USA). Band densities were quantified by Image-Pro Plus (version 6.0). The result was presented as the ratio of the optical density of the phosphorylated target band to the total target or the β-actin band.

### Detection of reactive oxygen species and mitochondrial membrane potential

Cells were grown on 6 well-culture plates at 3 × 10^5^ cells/well. The intervention was the same as before. Then, the medium was removed and then washed twice with PBS. To detect reactive oxygen species (ROS), DCFH-DA working solution was added and incubated for 60 min. After washing with twice PBS, cells were visualized by fluorescence microscopy (TE2000-E, Nikon). To detect the mitochondrial membrane potential (MMP), cells were incubated with JC-1 working solution (1 mL JC-1 working solution plus 1 mL complete DMEM in 6 well-culture plates) for 20 min, followed washing twice with pre-cold JC-1 staining buffer. Images were captured by the fluorescence microscopy.

### Statistical analysis

All results were presented as mean ± standard deviation (SD) and analyzed through SPSS 19.0 software. One-way analysis of variance (ANOVA) was used to determine the statistical significance. Based on whether data assumed equal variances or not, LSD or Dunnett’s T3 test was used, respectively. *P* < 0.05 was considered statistically significant.

## Results

### Cell model of NAFLD

**Fig. 1:**
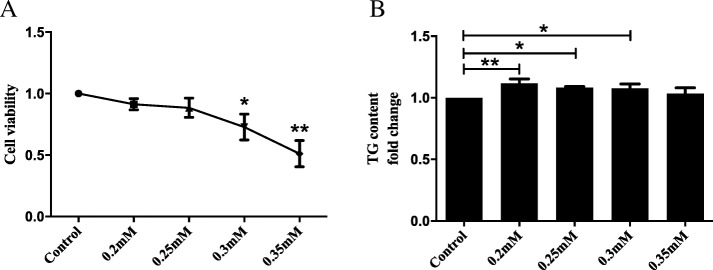
Cell model of NAFLD. **a**. PA was cytotoxic at concentrations> 0.25 mM. **b**. PA at 0.2 mM, 0.25 mM and 0.3 mM all induced significant lipid accumulation in LO2 cells. * *P* < 0.05 and ** *P* < 0.01

An in vitro NAFLD model was established by using PA. We incubated LO2 cells with culture medium containing PA (0.2–0.35 mM) for 24 h, and then determined the intracellular TG content to identify lipid overload. The CCK-8 assay was performed to evaluate the toxicity of PA. It was found that PA was cytotoxic at concentrations> 0.25 mM (Fig. 1a) and 0.2 mM, 0.25 mM, and 0.3 mM PA all induced significant lipid accumulation (Fig. 1b). Therefore, the maximum nontoxic concentration of PA (0.25 mM) was used for this study.

### DSG, A-769662, and compound C for cell viability

**Fig. 2:**
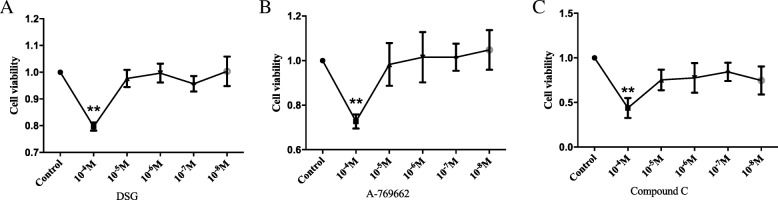
DSG, A-769662, and Compound C for cell viability. The maximum nontoxic concentration was as follows: **a**. DSG (10^− 5^ mol/L); **b**. A-769662 (10^− 5^ mol/L); **c**. Compound C (10^− 5^ mol/L). * *P* < 0.05 and ** *P* < 0.01

We screened the drug concentration before performing further experiments. The maximum nontoxic concentration was as follows: DSG (10^− 5^ mol/L), A-769662 (10^− 5^ mol/L), and Compound C (10^− 5^ mol/L) (Fig. 2a-c). Different noncytotoxic concentrations of DSG (10^− 7^ mol/L, 10^− 6^ mol/L and 10^− 5^ mol/L) were used in this study.

### The effect of DSG on intracellular lipid accumulation in LO2 cells

**Fig. 3:**
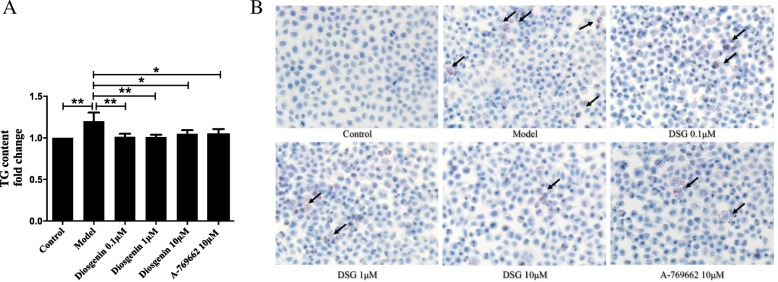
The effect of DSG on intracellular lipid accumulation in LO2 cells. **a**. DSG treatment decreased intracellular TG content in LO2 cells. **b**. Staining of lipid droplets with Oil Red O showed that PA caused an apparent increase in intracellular lipid accumulation, which was attenuated by the treatment with DSG (× 400 magnification). * *P* < 0.05 and ** *P* < 0.01

As shown in Fig. 3, the content of intracellular TG in the model group was markedly increased compared with control group (*P* = 0.0009), and DSG treatment significantly suppressed this increase (P_0.1μM_ = 0.0019, P_1μM_ = 0.0015 and P_10μM_ = 0.0144 vs model group) while the treatment with the AMPK activator A-769662 had the same result (*P* = 0.0102 vs model group), indicating the role of AMPK signaling in this process (Fig. 3a). Moreover, Oil Red O staining was used to evaluate the lipid accumulation in LO2 cells. Compared with control group, PA-treated cells had more lipid droplets, and DSG or A-769662 treatment all caused an obvious decrease in lipid droplets (Fig. 3b). These data suggested that both DSG treatment and AMPK signaling activation can reduce cellular lipid accumulation.

### The effect of DSG on PA-induced cell injury

**Fig. 4:**
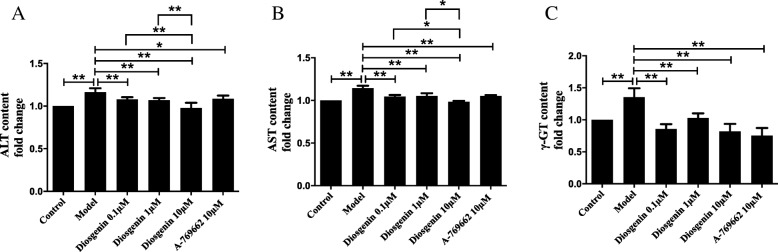
The effect of DSG on PA-induced cell injury. DSG treatment attenuated PA supplementation-induced release of ALT (**a**), AST (**b**) and γ-GT (**c**). * *P* < 0.05 and ** *P* < 0.01

Accompanied with lipid accumulation, PA supplementation increased the release of intracellular hepatic enzymes, such as ALT, AST and γ-GT (*P* < 0.01 vs control group) (Fig. 4a-c). Consistent with cellular lipid accumulation, treatment with DSG and A-769662 led to a decreased the enzyme content in the cell supernatant (*P* < 0.05 vs model group).

### The effect of DSG on cell glucose uptake

**Fig. 5:**
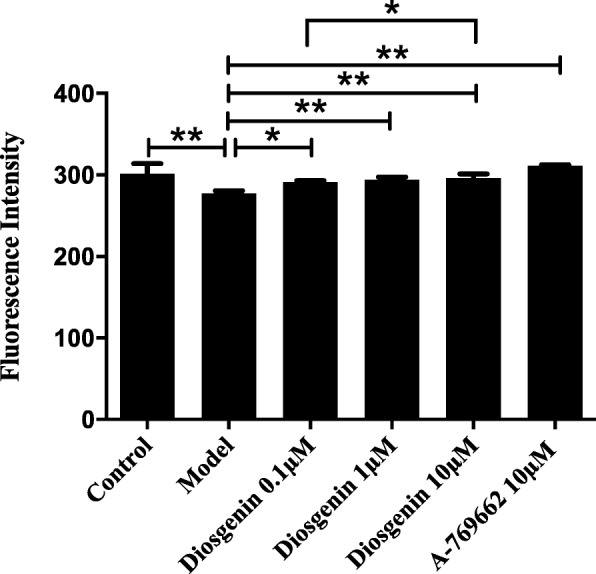
The effect of DSG on cell glucose uptake. DSG treatment attenuated impaired cell glucose uptake in LO2 cells. * *P* < 0.05 and ** *P* < 0.01

Given the close relationship between glucose and lipid metabolism, we investigated the cell glucose uptake in LO2 cells. Decreased glucose uptake was found in the model group compared with control group (*P* = 0.0002 vs control group), indicating the impaired glucose utilization. As expected, cells showed a significant increase in glucose uptake after treatment with DSG (P_0.1μM_ = 0.0356, P_1μM_ = 0.0091 and P_10μM_ = 0.0026 vs model group) or A-769662 (*P* < 0.0001 vs model group) (Fig. 5).

### The effect of DSG on mitochondrial function

**Fig. 6:**
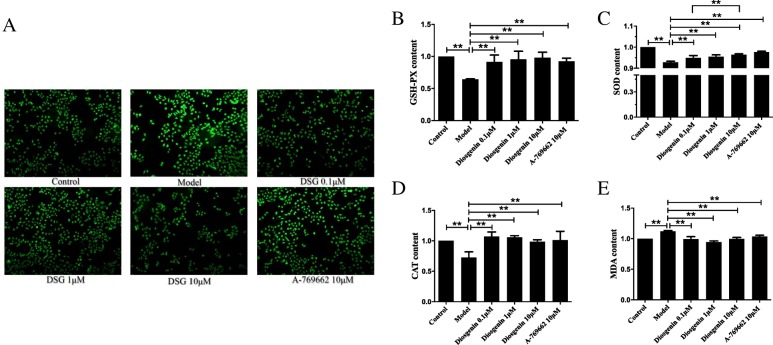
The effect of DSG on oxidative stress. **a**. DSG treatment can significantly reduce the production of ROS (× 200 magnification) and increase the antioxidant enzymes, including GSH-PX (**b**), SOD (**c**) and CAT (**d**), compared with model group. **e**. DSG treatment significantly reduced the content of MDA. * *P* < 0.05 and ** *P* < 0.01

Mitochondria are the central site of lipid catabolism, and their dysfunction plays a crucial role in the pathogenesis of NAFLD [23]. To verify whether improved mitochondrial activity is involved in the effect of DSG on PA-induced lipid accumulation in LO2 cells, we examined the mitochondrial function. Compared with control group, the ROS and MDA contents in model group were remarkably increased (*P* = 0.0003 vs control group), suggesting the excessive oxidative stress in PA-treated cells. Treatment with DSG or A-769662 significantly reduced the ROS and MDA contents (*P* < 0.01 vs model group) (Fig. 6a and e). Meanwhile, the antioxidant enzymes including GSH-PX, SOD and CAT, were downregulated in model group (*P* < 0.01 vs control group), but the DSG groups and A-769662 group had increased contents of these antioxidant enzymes compared with model group (*P* < 0.01 vs model group) (Fig. 6b-d). These results indicated that DSG improved mitochondrial dysfunction by inhibiting oxidative stress in PA-treated cells.

**Fig. 7:**
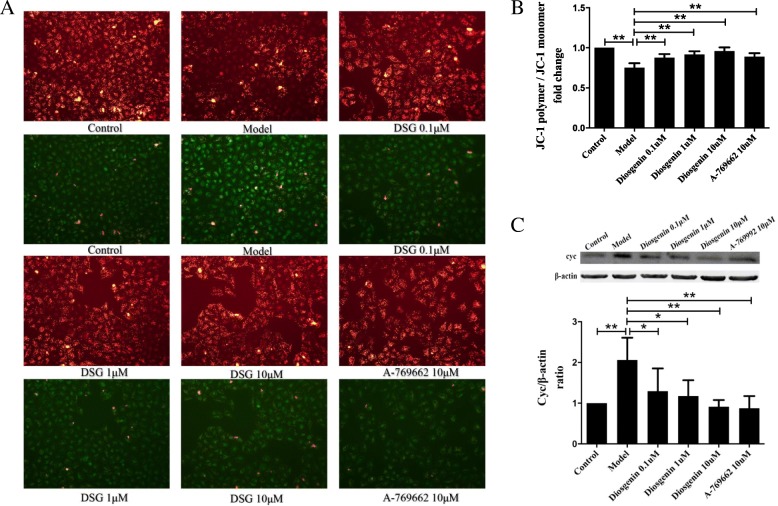
The effect of DSG on mitochondrial membrane potential and cytochrome c. **a**. Representative micrographs (× 200 magnification) of JC-1 showed that DSG treatment was associated with a significant increase in MMP. **b**. The ratio of JC-1 polymer/ JC-1 monomer from each group. **c**. Effects of DSG on the expression of cyc. * *P* < 0.05 and ** *P* < 0.01

JC-1, a dual-emission potential-sensitive probe was used to measure the mitochondrial membrane potential (MMP). At higher potentials, JC-1 forms the red-fluorescent “JC-1 polymer” which converts to the green-fluorescent “JC-1 monomer”. Cytochrome c (Cyc) is released from the mitochondrial inner membrane into the cytoplasm when mitochondria are damaged. Studies have confirmed that changes occur of the MMP and Cyc in mitochondrial dysfunction. As shown in Fig. 7, PA-treated cells exhibited an obvious decrease in the MMP with lower red/green ratio (*P* = 0.0001 vs control group) and more cytoplasmic Cyc (*P* = 0.0015 vs control group) compared with control group (Fig. 7a and b). Then, treatment with either DSG or AMPK activator A-769662 led to a significant increase in MMP (P_0.1μM_ = 0.0005, P_1μM_ = 0.0001, P_10μM_ < 0.0001 and P_A-769662_ = 0.0007 vs model group) and less cytoplasmic Cyc leakage (P_0.1μM_ = 0.012, P_1μM_ = 0.0105, P_10μM_ = 0.0005 and P_A-769662_ = 0.0001 vs model group) (Fig. 7c).

### The effect of DSG on the AMPK/ACC/CPT-1A and SREBP-1c/FAS signaling pathway

**Fig. 8:**
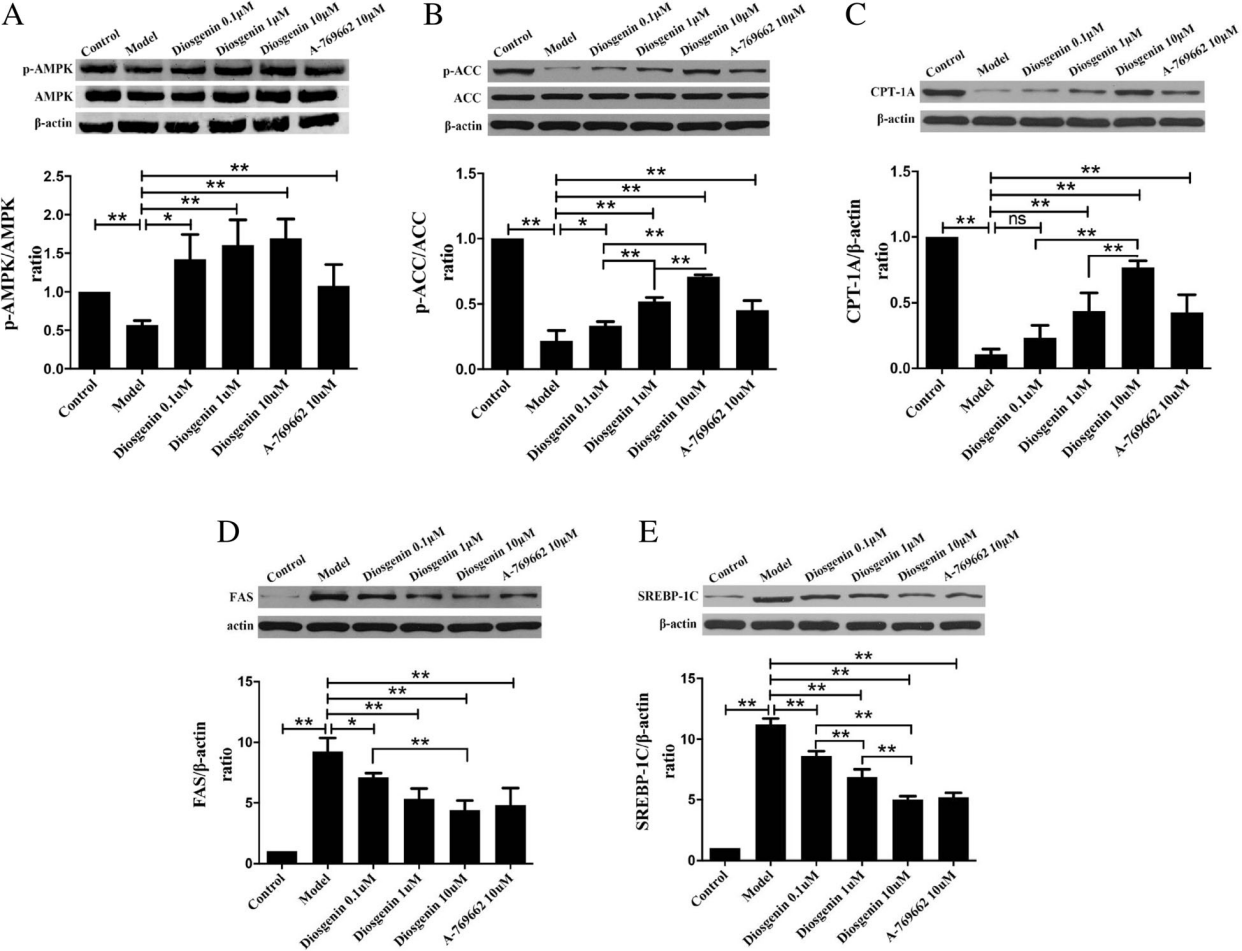
The effect of DSG on AMPK/ACC/CPT-1A and SREBP-1c/FAS signaling pathway. Western blot analysis of protein expression of AMPK/ACC/CPT-1A and SREBP-1c/FAS signaling pathway, AMPK and p-AMPK (**a**), ACC and p-ACC (**b**), CPT1A (**c**), SREBP-1c (**d**) and FAS (**e**). * *P* < 0.05 and ** *P* < 0.01

**Fig. 9:**
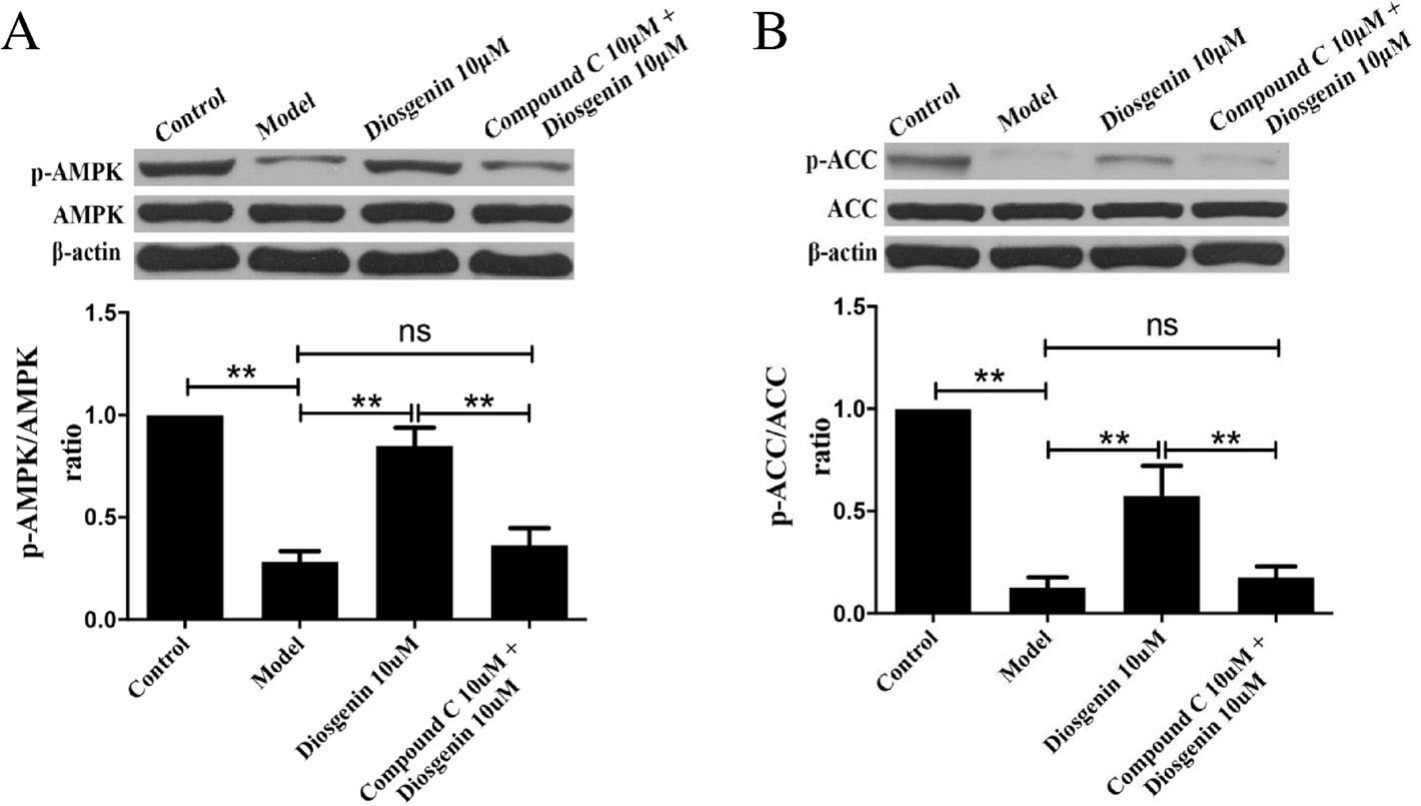
The effect of DSG on AMPK/ACC signaling pathway after AMPK inhibitor was pre-administered. Western blot analysis of protein expression of AMPK/ACC signaling pathway, AMPK and p-AMPK (**a**), ACC and p-ACC (**b**). * *P* < 0.05 and ** *P* < 0.01

**Fig. 10:**
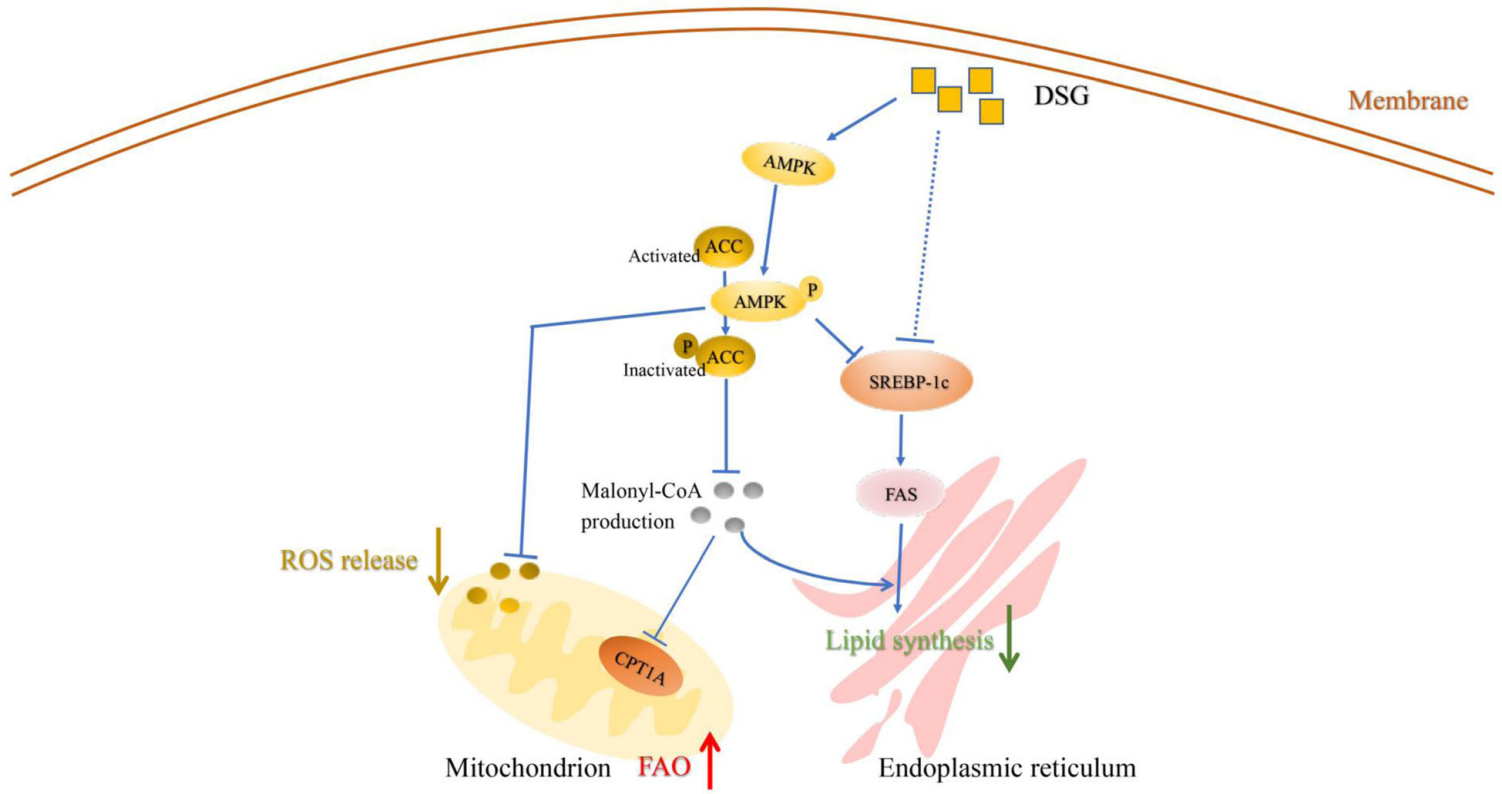
Diosgenin ameliorates palmitic acid-induced lipid accumulation in LO2 cells and that the underlying mechanism may be related to the improving oxidative stress, increasing fatty acid β-oxidation and decreasing lipid synthesis

In order to explore the mechanism of DSG on ameliorating lipid accumulation in LO2 cells, we examined the protein expression of p-AMPK, AMPK, p-ACC, ACC, CPT1A, SREBP-1c, FAS and β-actin. In our study, compared with model group, the protein expression of p-AMPK, p-ACC and CPT1A was significantly increased (*P* < 0.05 vs model group) and that of SREBP-1c and FAS was decreased (*P* < 0.05 vs model group) in the DSG groups and A-769662 group (Fig. 8a-e). These data suggested that the possible mechanisms of DSG were associated with the activation of the AMPK/ACC/CPT-1A pathway and inhibition of the SREBP-1c/FAS pathway. For further verification, we performed another experiment using the AMPK inhibitor Compound C. Compound C was preincubated with LO2 cells for 5 h to inhibit the activation of AMPK in advance, and then, LO2 cells were treated with PA and DSG for another 24 h. The protein expression levels of p-AMPK and p-ACC were detected by western blot. As expected, Compound C preadministration eliminated the enhanced activity of DSG on the AMPK/ACC signaling (Fig. 9a and b). The contents of p-AMPK and p-ACC in inhibitor group were significantly reduced (P _p-AMPK_ < 0.0001 and P _p-ACC_ = 0.0016 vs DSG group) compared with DSG group and showed no difference with model group (P _p-AMPK_ = 0.589 and P _p-ACC_ = 0.8816 vs model group). These results suggested that the anti-hepatic steatosis effect of DSG may be related to its direct activation of the AMPK/ACC/CPT-1A pathway. Taken together, these findings demonstrated that the underlying mechanism of DSG on ameliorating hepatic lipid accumulation was associated with direct activation of the AMPK/ACC/CPT-1A signaling pathway to increase lipolysis and inhibition of the SREBP-1c/FAS signaling to reduce lipid synthesis (Fig. 10).

## Discussion

Hepatic lipid accumulation is considered as the key loop in the pathogenesis of NAFLD and can be caused by multiple factors, including increased uptake of free fatty acids into the liver, impaired fatty acid β-oxidation, or the increased incidence of de novo lipogenesis [4, 24, 25]. It has been shown that hepatic lipid accumulation is linked with insulin resistance, mitochondrial stress, impaired autophagy and endoplasmic reticulum stress (ER stress), eventually leading to the progression of liver injury [5], which in turn, contributes to lipid overload. Thus, reducing lipid accumulation in the liver is an important and effective approach for treating NAFLD. As previously mentioned, based on the mechanism of lipid metabolism, accelerating lipid consumption and inhibiting lipid synthesis are alternative strategies to decrease the lipid content.

Mitochondria are the power producer of the cell, which plays a central role in the generation of energy from nutrient oxidation [26]. Numerous studies have indicated that mitochondrial dysfunction contributes to the pathogenesis of NAFLD since it affects hepatic lipid homeostasis [27]. Under the “first hit” (such as insulin resistance, hyperlipidemia), increased FFA flux occurs in the liver. If FFAs are not oxidized or secreted, hepato-steatosis develops [28]. It has been reported that the accumulation of toxic lipid intermediates in the liver leads to the production of ROS when the excessive content of lipids exceeds the cell’s catabolic capacity [29]. Under normal conditions, most mitochondrial ROS are usually detoxified by antioxidant enzymes, whereas overproduction of ROS induces oxidative stress [30]. These changes may damage mitochondrial proteins, lipids and mitochondrial DNA, and lead to mitochondrial dysfunction with decreased MMP and increased cytoplasmic Cyc, ultimately causing cellular dysfunction and death; these changes are in accordance with our data. Interestingly, there is a hypothesis is that a “primary” mitochondrial dysfunction may be the initiator of lipid accumulation in the hepatocytes [31]. The “primary” mitochondrial dysfunction impairs the lipid homeostasis and gradually leads to hepatic steatosis. Whether they are the cause or the result, mitochondrial dysfunction and lipid overload form a vicious circle and contribute to each other. Hence, improving mitochondrial dysfunction become a useful therapeutic approach for reducing lipid overload. Several studies have shown that antioxidant products supplementation can protect against high-fat diet-induced NAFLD [32, 33], which is consistent with our results. In this study, we explored the effects of DSG on oxidative stress and mitochondrial dysfunction. Previous studies have confirmed that DSG has antioxidant activity [34], which was also observed in our work. More importantly, we found that impaired mitochondrial function was obviously ameliorated by DSG treatment. Although DSG has been shown to have a wide range of pharmacological activities in many studies [35], our work attributed its improvement of mitochondrial dysfunction to its antioxidant activity.

Notably, A-769662, an AMPK activator, has also been shown to have antioxidant activity and improved mitochondrial dysfunction, similar to DSG. The activation of AMPK protects cells from oxidative stress, which has been well identified by a large number of studies of metformin [36], one of the most widely used AMPK activators. Several lines of evidence suggest that AMPK prevents oxidative stress mainly by regulating glucose and lipid metabolism. In this work, we postulate that DSG exerts its antioxidant activity in part by activating AMPK. Further investigations are needed to determine whether DSG can protect against oxidative stress via a non-AMPK or nonmetabolism pathway.

Depending on the physiological conditions, increased FFAs have multiple destinations in the liver: (1) FFAs are converted into complex lipid species, packaged into very low density lipoproteins (VLDL) and released into the circulation; (2) oxidized by β-oxidation; or (3) esterified into triglyceride (TG) and stored as lipid droplets surrounded by lipid droplet proteins within the hepatocyte [37]. Clearly, fatty acid β-oxidation receives more attention in regard to the disposal of hepatic FFAs because the other two methods do not reduce lipid overload. As a cellular energy sensor, AMPK signaling is regarded as one of the most important regulators of lipid homeostasis [38]. Increasing evidence has shown that activation of AMPK signaling can promote lipid catabolism by stimulating FAO and inhibiting lipogenesis [3, 39]. As demonstrated above, once activated, AMPK induces the inhibitory phosphorylation of ACC. ACC is a key enzyme in fatty acid synthesis that can catalyze the carboxylation of acetyl-CoA to malonyl-CoA. Thus, the activation of AMPK causes a decrease in cellular malonyl-CoA levels, which is an endogenous inhibitor of CPT1, ultimately stimulating FAO [12, 40]. Additionally, lipogenesis is directly limited by the reduction of malonyl-CoA, which is also an important precursor of lipid biosynthesis. Therefore, AMPK activators have been proven to be effective in lipid disorders [41]. That was also confirmed by our work, in which the AMPK activator A-769662 was shown to have a significant effect on reducing lipid accumulation. Many studies have shown that DSG and its analogs can activate the AMPK pathway to prevent the development of NAFLD [22, 42, 43]. Our data were in accordance with these published works, and DSG remarkably increased the p-AMPK content as same as A-769662. However, the effect of DSG on FAO via the AMPK/ACC/CPT-1A pathway has not been investigated. Our results indicated that DSG treatment significantly improved AMPK activity, inhibited ACC activity and, eventually, increased CPT-1A function. The enhanced FAO in DSG-treated cells was mediated by the AMPK/ACC/CPT-1A pathway.

The transcription factor SREBP-1c is a master regulator of lipogenesis, which is involved in the transcriptional activation of genes encoding rate-limiting enzymes in the lipogenesis, such as FAS, ACC and stearoyl-CoA desaturase 1 (SCD1). It has been reported that SREBP-1c activation is associated with increased lipogenesis in NAFLD [17, 24]. On the contrary, liver-specific SREBP-1c knockout (KO) mice have a lower hepatic lipid content as well as lower plasma TG levels compared to their wild-type control [44]. Consistent with ACC, the activity of SREBP-1c is negatively regulated by AMPK [3, 43], which directly phosphorylates SREBP-1c and inhibits it cleavage and nuclear translocation. AMPK suppresses SREBP-1c expression, which reduces FAS expression and, subsequently, inhibits lipid synthesis. Similarly, AMPK activator A-769662 significantly decreased the expression of SREBP-1c and FAS in our study. In addition to direct regulation, recent studies have revealed that AMPK can suppress SREBP-1c expression by decreasing the activity of mammalian target of rapamycin complex (mTORC) [45], an important mediator for the regulation of cellular metabolism and growth that is thought to accelerate fatty acid synthesis by cleavage of the SREBP-1c molecule [46]. Moreover, activation of AMPK may also suppress the transcriptional activity of downstream targets, such as liver X receptors (LXRs), which are an upstream transcription factors that increase SREBP-1c activity to promote fatty acid synthesis [47, 48]. Altogether, the DSG-induced AMPK activation inhibits SREBP-1c signaling through various mediators. However, whether DSG can directly inhibit the SREBP-1c activity is not fully clear, and some studies have shown that DSG can inhibit the transcription of *srebp-1c* [42]; however, there is still a lack of evidence that DSG directly inhibits SREBP-1c activity. Diosgenin is structurally related to cholesterol and has been used as a precursor for steroidal hormone synthesis [35]. Therefore, we believe that more studies are needed in this field to study the interaction between DSG and SREBP-1c. Moreover, improved glucose uptake was observed in DSG groups, which might be related to an increase of the protein expression of glucose transport-4 (GLUT-4) and its membrane translocation according to our previous study [21].

## Conclusions

In conclusion, we demonstrated that DSG reduces the PA-induced lipid accumulation in LO2 cells. The underlying mechanism may be related to improving oxidative stress, increasing fatty acid β-oxidation and decreasing lipid synthesis. Molecular studies revealed that the anti-NAFLD effect of DSG might be mediated by the activation of the AMPK/ACC/CPT-1A pathway and inhibition of the SREBP-1c/FAS pathway.

## Data Availability

The data used and/or investigated during the present study are available from the corresponding author upon reasonable request.

## Abbreviations

ACC: Acetyl-coA carboxylase
AMPK: AMP activated protein kinase
CPT-1A: Carnitine acyl transferase 1A
Cyc: Cytochrome c
DSG: Diosgenin
FAO: fatty acid β-oxidation
FAS: Fatty acid synthase
GLUT-4: Glucose transporter-4
LO2 cells: Human normal hepatocytes
LXRs: liver X receptors
MMP: Mitochondrial membrane potential
mTORC: mammalian target of rapamycin complex
NAFLD: Nonalcoholic fatty liver disease
SCD1: stearoyl-CoA desaturase 1
SREBP-1c: Sterol regulatory element-binding protein 1c
TG: triglyceride
VLDL: very low density lipoproteins
